# Supplementary material for: Increased Utilization of Abdominal Surgical Procedures, Endoscopy and Imaging After Negative Rectal Biopsies for Suspected Hirschsprung’s Disease: A Danish Nationwide Matched Cohort Study
Source: Children (Basel). 2025 Aug 24;12(9):1112. doi: 10.3390/children12091112 (PMC12468526; doi:10.3390/children12091112)
Supplement: Supplementary file 1 [file children-12-01112-s001.zip › children-3796012-supplementary.pdf]

Supplementary material:

Table S1. Included procedure codes classified by NSCP (Nordic Classification of Surgical Procedures)

Category	NOMESCO Codes	Description
Surgical Procedures	KA01*, KB01*, KC01*, KD01*, KE01*, KF01*, KG01*, KH01*, KJ01*, KK01*, KL01*, KM01*, KN01*, KP01*, KQ01*, KT01*, KU01*, KW01*, KX01*	All abdominal and general pediatric surgical procedures (e.g., appendix, intestines, hernia, urogenital)
Radiological Examinations	UXA01*, UXC01*, UXM01*, UXR01*, UXU01*	Abdominal radiological imaging (US, CT, MRI, X-ray, fluoroscopy)
Endoscopic procedures	KUJD*, KUJF*	Upper and lower endoscopic examinations

Table S2. Univariate and multivariate logistic regression analyses for factors associated with surgery after index date (ID). Non-HD compared to control group. Odds ratios (OR) and 95% confidence intervals (CI). BMI= body mass.

Variable	Univariate OR (95% CI)	P-value	Multivariate OR (95% CI)	P-value
Group				
Non-HD	7.82 (6.10-10.03)	<0.001	4.93 (3.46-6.99)	<0.001
Control	1.00 (base)	-	1.00 (base)	-
Sex				
Female	1.00 (base)	-	1.00 (base)	-
Male	1.45 (1.14-1.85)	0.002	1.45 (1.12-1.87)	0.004
Gestational age	0.98 (0.97-0.98)	<0.001	0.99 (0.98-0.99)	<0.001
Length at birth	0.97 (0.96-0.99)	<0.001	1.01 (0.99-1.03)	0.462
APGAR score at 5 min	0.86 (0.75-0.99)	0.034	0.99 (0.84-1.17)	0.907
Mother's BMI	1.01 (1.00-1.02)	0.145	-	-
Mother smoking				
No	1.00 (base)	-	1.00 (base)	-
Yes	1.60 (1.21-2.10)	0.001	1.40 (1.04-1.88)	0.027
Equivalated family income	0.99 (0.99-0.99)	0.038	1.00 (0.99-1.00)	0.154
Suurgery befor ID				
No	1.00 (base)	-	1.00 (base)	-
Yes	9.17 (8.52-12.88)	<0.001	2.65 (1.76-3.99)	<0.001
Endoscopy before ID				
No	1.00 (base)	-	1.00 (base)	-

Variable	Univariate OR (95% CI)	P-value	Multivariate OR (95% CI)	P-value
Yes	7.58 (4.24-13.53)	<0.001	1.69 (0.87-3.27)	0.119
Imaging before ID				
No	1.00 (base)	-	1.00 (base)	-
Yes	4.80 (3.71-6.19)	<0.001	1.19 (0.82-1.72)	0.354

Table S3. Univariate and multivariate regression analysis for factors associated with the likelihood of undergoing endoscopy after index date (ID). Odds ratios (OR) with 95% confidence intervals (CI). BMI = body mass index.

Variable	Univariate OR (95% CI)	P-value	Multivariate OR (95% CI)	P-value
Group				
Non-HD	20.10 (14.34–28.16)	<0.001	10.89 (6.80–17.45)	<0.001
Control	1.00 (base)	-	1.00 (base)	-
Sex				
Female	1.00 (base)	-	1.00 (base)	-
Male	0.70 (0.51–0.97)	0.032	0.69 (0.49–0.98)	0.037
Gestational age	0.98 (0.97–0.99)	<0.001	1.00 (0.99–1.00)	0.758
Length at birth	0.97 (0.95–0.98)	<0.001	0.99 (0.97–1.01)	0.343
APGAR score at 5 min	0.84 (0.71–0.99)	0.041	0.99 (0.80–1.23)	0.932
Mother’s BMI	1.01 (0.99–1.02)	0.458	-	-
Mother smoking				
No	1.00 (base)	-	-	-
Yes	1.32 (0.89–1.97)	0.173	-	-
Equivalated family income	1.00 (0.99–1.00)	0.410	-	-
Surgery before ID				
No	1.00 (base)	-	1.00 (base)	-
Yes	9.35 (6.02–14.53)	<0.001	1.47 (0.87–2.49)	0.148
Endoscopy before ID				
No	1.00 (base)	-	1.00 (base)	-
Yes	23.05 (13.54–39.25)	<0.001	4.21 (2.28–7.77)	<0.001
Imaging before ID				
No	1.00 (base)	-	1.00 (base)	-
Yes	10.62 (7.66–14.73)	<0.001	1.70 (1.07–2.71)	0.026

Table S4. Univariate and multivariate logistic regression analyses of factors associated with the likelihood of undergoing imaging after the index date. (ID) Results are presented as odds ratios (OR) with 95% confidence intervals (CI). BMI = body mass index.

Variable	Univariate OR (95% CI)	P-value	Multivariate OR (95% CI)	P-value
Group				
Non-HD	18.23 (14.49–22.94)	<0.001	9.47 (6.89–13.01)	<0.001
Control	1.00 (base)	-	1.00 (base)	-
Sex				
Female	1.00 (base)	-	-	-
Male	0.97 (0.78–1.21)	0.776	-	-
Gestational age	0.98 (0.97–0.98)	<0.001	0.99 (0.98–0.99)	0.008
Length at birth	0.97 (0.96–0.98)	<0.001	1.00 (0.98–1.02)	0.877
APGAR score	0.88 (0.77–1.00)	0.075	-	-
Mother's BMI	1.01 (1.00–1.02)	0.152	-	-
Mother smoking				
No	1.00 (base)	-	1.00 (base)	-
Yes	1.36 (1.04–1.79)	0.023	1.19 (0.89–1.60)	0.239
Equivalated family income	1.00 (0.99–1.00)	0.897	-	-
Surgery before ID				
No	1.00 (base)	-	1.00 (base)	-
Yes	8.37 (6.02–11.64)	<0.001	1.51 (1.02–2.22)	0.039
Endoscopy before ID				
No	1.00 (base)	-	1.00 (base)	-
Yes	13.10 (8.13–21.12)	<0.001	2.23 (1.30–3.84)	0.004
Imaging before ID				
No	1.00 (base)	-	1.00 (base)	-
Yes	10.19 (8.13–12.76)	<0.001	1.93 (1.40–2.67)	<0.001
